# Self-Extinguishing Alginate-Based Xerogel Foams for Thermal Insulation

**DOI:** 10.3390/gels12070625

**Published:** 2026-07-11

**Authors:** Radmila Damjanović, Marija M. Vuksanović, Milena Stavrić, Jovana Ružić, Irena Živković, Radmila Jančić Heinemann

**Affiliations:** 1 Faculty of Applied Arts, University of Arts in Belgrade, 11000 Belgrade, Serbia; irena.zivkovic@fpu.bg.ac.rs; 2 VINČA Institute of Nuclear Sciences—National Institute of the Republic of Serbia, University of Belgrade, 11351 Belgrade, Serbia; jruzic@vin.bg.ac.rs; 3 Institute of Architecture and Media, Faculty of Architecture, Graz University of Technology, 8010 Graz, Austria; mstavric@tugraz.at; 4 Faculty of Technology and Metallurgy, University of Belgrade, 11000 Belgrade, Serbia; radica@tmf.bg.ac.rs

**Keywords:** insulation, xerogel, alginate, expanded perlite, self-extinguishing, eco-friendly, composite foam

## Abstract

Biopolymer-based porous materials are attracting increasing interest as sustainable alternatives to conventional thermal insulation foams; however, achieving low thermal conductivity together with adequate mechanical performance and fire response remains challenging. Building on previous formulation screening, this study investigates alginate-expanded perlite xerogel foams modified with chitosan and glycerol for thermal insulation applications. Foams were prepared via in situ CO_2_ foaming and Ca^2+^ crosslinking, followed by mild oven drying. The effects of expanded perlite (9–12%), glycerol (0–10%), and chitosan (0–1%) were systematically investigated. All formulations exhibited low thermal conductivity (0.0467–0.0525 W m^−1^ K^−1^) and UL-94 V-0 self-extinguishing behavior. Incorporation of dispersed chitosan significantly enhanced compressive strength, reaching 263 kPa at 10% strain, within the range of commercial polymer foams. Image analysis and SEM showed that chitosan suppressed bubble coalescence, reduced large-pore fractions, and improved particle coverage, yielding structurally coherent matrices. Glycerol primarily acted as a plasticizer, improving the dimensional stability, but contributing less to pore refinement. The developed foams combine competitive thermal insulation performance, self-extinguishing behavior, and mechanical properties suitable for self-supporting insulation applications through a simple, water-based manufacturing route, highlighting their potential as sustainable alternatives to conventional fossil-based insulation materials for building applications.

## 1. Introduction

The development of sustainable thermal insulation materials increasingly focuses on replacing conventional synthetic foams with biobased alternatives that offer improved fire safety and environmental performance [1]. Widely used insulators, such as expanded polystyrene and polyurethane, exhibit low thermal conductivity (~0.02–0.04 W m^−1^ K^−1^) [2]; however, their fossil origin, flammability, and end-of-life challenges raise significant concerns [3,4,5,6]. Consequently, research has shifted toward biopolymer-based systems that can provide competitive insulation performance while offering sustainability and enhanced flame-retardant properties [7,8].

Sodium alginate, an anionic polysaccharide derived from brown algae [9] represents a promising matrix material for porous insulation materials due to its water-based processability [10], ion-induced gelation [11], non-toxicity, and biodegradability [12]. When crosslinked with polyvalent ions such as Ca^2+^, alginate exhibits enhanced thermal stability and flame retardancy, which is primarily associated with protective char formation [13,14,15,16,17,18]. Nevertheless, pure alginate foams require additional reinforcement to achieve the mechanical robustness necessary for building applications, due to their inherently low mechanical strength [19]. A key challenge lies in balancing low density with sufficient mechanical strength, since such materials often exhibit low resistance at small deformations. In many studies, the mechanical performance of alginate-based porous materials is evaluated at higher compressive strains, where the densification of cellular structure dominates the stress–strain response, providing insight into structural collapse and mechanical stability [14,15,19,20]. However, for insulation applications, materials are generally subjected to relatively low service strains, making stress at 10% strain a more relevant parameter, consistent with standards used for conventional insulation materials such as expanded and extruded polystyrene (EN 13163 and EN 13164, respectively) [21,22]. Evaluating compressive performance in this regime enables meaningful comparison with existing insulation systems and better reflects service conditions.

To address the mechanical limitations of alginate foams, modification of the alginate matrix is required. In this work, chitosan is introduced into the alginate matrix as a dispersed solid component. It is a non-toxic and biodegradable product [23], derived from chitin, that strengthens alginate structures [9]. Unlike conventional methods, where chitosan is predissolved in acidic media, its introduction in powder form facilitates a controlled expansion and enhances the matrix continuity. In this form, chitosan acts as a structural modifier that contributes to the stabilization of the expanding cellular structure during formation.

Further enhancement is achieved by combining the biopolymer matrix with expanded perlite reinforcement, a porous aluminosilicate material [24], known for its lightweight structure, non-toxicity [25], and excellent thermal and fire resistance [26]. It is commonly used in bulk form, enclosed within the construction panels [27], or in insulation boards with binders like starch and soluble alkali silicates [28,29,30,31]. While alginate-expanded perlite systems have been explored primarily as beads/particles for encapsulation and adsorption [32,33,34], their application as foamed insulation composites remains largely unexplored. Foaming can be achieved via CO_2_ formation from sodium bicarbonate and acetic acid. In previous work [35], this approach enabled the preparation of alginate xerogel foams with various biopolymer additives, among which chitosan and glycerol showed the most favorable influence on mechanical performance. Chitosan was selected due to its reinforcement role, as a chitosan-only containing formulation resulted in the highest tensile strength value (120 kPa), while chitosan-containing formulations generally exhibited higher elastic modulus values than their chitosan-free counterparts [35]. Glycerol, a biodegradable and non-toxic polyol [36], was selected because it has a well-established role as a plasticizer for sodium alginate, and it has been reported that it can improve the flexibility and reduce brittleness of the alginate matrix [37]. In the previous screening study [35], the formulation with 15 wt.% glycerol reached the highest toughness (19.1 kJ m^−3^), while retaining relatively high tensile strength (111 kPa). This result was used to identify glycerol as a relevant plasticizing additive in the screening stage, rather than to define glycerol content in the present study. Accordingly, the present work further examines the effect of chitosan and glycerol within selected alginate-expanded perlite xerogel formulations.

Beyond mechanical performance, thermal stability and fire resistance are critical requirements for building insulation materials. Biopolymer materials such as alginate can contribute to fire resistance through char formation during thermal degradation [38]. The presence of inorganic components and multivalent cations such as Ca^2+^ promotes the formation of carbonaceous and mineral residues that form a dense insulating barrier, reducing heat transfer and inhibiting further degradation [15]. Expanded perlite, as a thermally stable and non-combustible component, additionally supports the fire resistance of the composite systems.

A further limitation in the development of biobased porous insulation materials is the frequent reliance on freeze-drying [7]. It is an energy-intensive and costly process, with an energy consumption significantly exceeding that of conventional hot-air drying (reported in the range of approximately 4–10 times) [39,40]. In addition to energy and cost restraints, the industrial translation of biobased porous insulation materials is often limited by inherent mechanical fragility under compressive and bending loads [41] and the sensitivity of pore structure to freezing conditions, which strongly influences pore size and shape [42]. These factors complicate reproducibility and large-area fabrication. Therefore, simpler processing routes are needed to produce thermally efficient and mechanically stable biobased insulation materials.

Unlike many biobased foams produced via freeze-drying, this study develops a straightforward in situ CO_2_ foaming approach combined with mild ambient-pressure drying to obtain xerogel foams. All materials employed in the preparation are abundant and affordable, as they are widely available biopolymers and commodity chemicals [25,43,44,45,46,47,48,49]. Non-toxic materials constitute the structural components of the system, while low-toxicity reagents are involved only transiently during the foaming and crosslinking steps [50,51,52]. Water is used as the sole processing solvent. The objective was to evaluate the influence of expanded perlite, chitosan, and glycerol on pore structure, thermal conductivity, tensile and compressive performance, and response to flame exposure. Particular attention was devoted to compressive performance at 10% strain, enabling comparison with conventional thermal insulation materials used in building applications. The innovation of this work lies in the synergistic design of the chitosan-enriched alginate matrix with expanded perlite, yielding a self-extinguishing (UL-94 V-0) material with low thermal conductivity. By eliminating cost- and energy-intensive drying protocols and relying instead on simple, ambient-pressure processing conditions, this method is inherently compatible with scalable manufacturing routes for environmentally friendly insulation materials.

## 2. Results and Discussion

### 2.1. Diameter Shrinkage and Apparent Density

**Figure 1:**
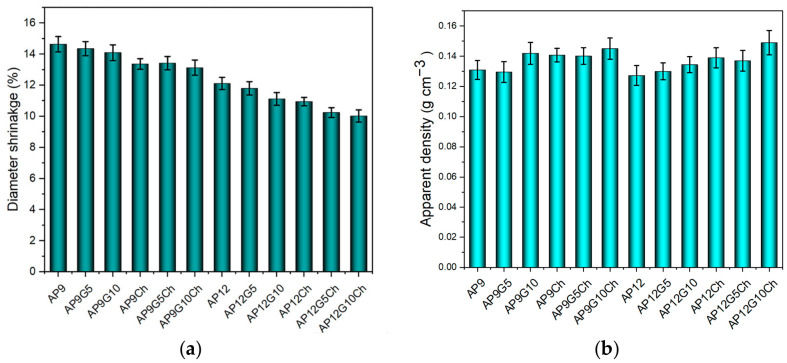
(**a**) Diameter shrinkage, and (**b**) apparent density of tested specimen formulations.

All specimens exhibited a measurable decrease in diameter after the removal from the CaCl_2_ bath and subsequent drying, with shrinkage ranging from 10.0% to 14.6% (Figure 1a). Higher perlite content consistently resulted in lower shrinkage, indicating its role as a dimensionally stable reinforcement that limits structural contraction. The addition of chitosan further reduced shrinkage by reinforcing polymer walls and limiting contractions during the dehydration process, while glycerol improved dimensional stability through its plasticizing effect, reducing chain packing during drying [37]. Apparent density values ranged from 0.124 to 0.149 g cm^−3^ (Figure 1b) and increased with the addition of glycerol and chitosan across both expanded perlite series (9 wt.% and 12 wt.%).

### 2.2. Surface Pore Ratio and Pore Size Distribution

**Figure 2:**
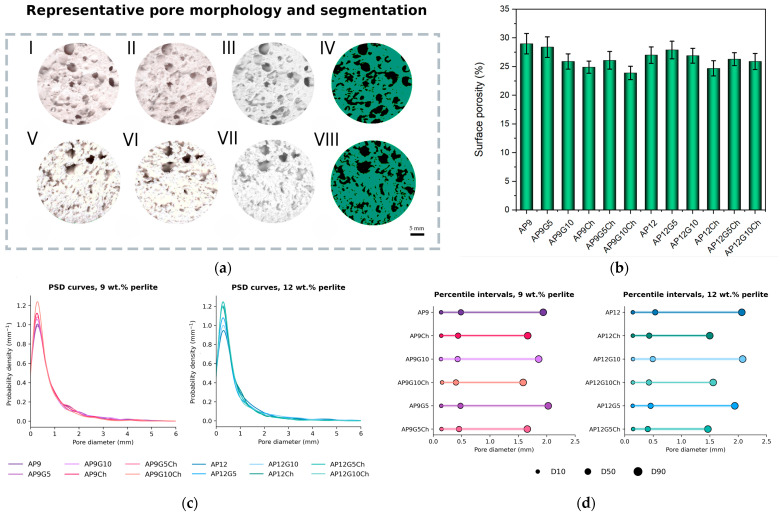
Pore morphology, surface porosity, and pore size distribution of alginate-perlite foams. (**a**) Surface scans of AP9 under different light conditions (I, II), followed by the fused (III) and segmented (IV) image, and surface scans of AP12G5Ch (V, VI) with the corresponding fused (VII) and segmented (VIII) image. (**b**) Surface porosity of tested specimens. (**c**) Probability density functions of pore size distribution for formulations containing 9 wt.% and 12 wt.% perlit. (**d**) Pore diameters (D10, D50, D90) for 9 wt.% and 12 wt.% formulations.

Due to variations in specimen diameter caused by shrinkage, all scanned specimen images were circularly cropped to a uniform size prior to analysis (Figure 2a). The mean surface pore ratio ranged from 23.9 to 29.0% (Figure 2b). Measurements were conducted on three independent replicates per formulation. Chitosan-free specimens exhibited higher surface porosity compared to those containing chitosan.

The pore size data extracted from segmented images were statistically summarized in Python 3.8.17; pore size distribution (PSD) curves were generated by Gaussian kernel density estimation. The percentile descriptors D10, D50, and D90 correspond to the pore diameters below which 10%, 50%, and 90% of the measured pores are found, respectively. The PSD curves of representative formulations (Figure 2c) are right-skewed, indicating a predominance of small pores, while larger pores are present only as a minor fraction. The percentile plots (Figure 2d) show that the main differences between formulations are most pronounced at higher pore diameters, particularly D90.

The effects of glycerol and chitosan on pore size differ. Increasing glycerol content (5–10 wt.%) results in minor, formulation-dependent changes, without a consistent trend in D50 or D90 and minimal effect on D10. In contrast, the presence of chitosan leads to a more pronounced reduction in D50 and D90, indicating suppression of larger pore formation.

Factorial ANOVA performed on the original pore size descriptors showed that chitosan was the only significant factor affecting D50 and D90, with the strongest effect observed for D90. No significant effect was found for D10. The same trend was obtained for log-transformed pore size descriptors. All reported effects were significant at *p* < 0.05.

### 2.3. FTIR Characterization

**Figure 3:**
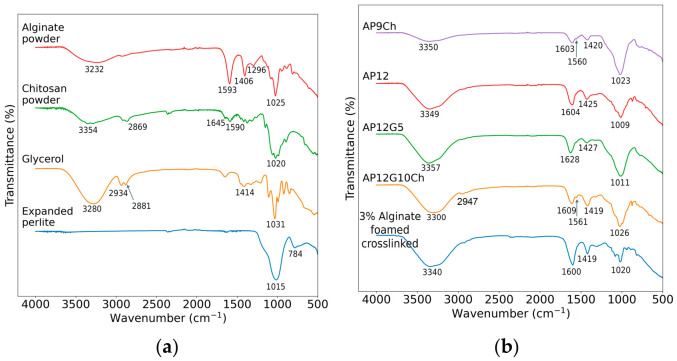
FTIR spectra of (**a**) raw materials, (**b**) selected specimens.

Figure 3a shows the FTIR spectra of raw material powders, and Figure 3b shows the FTIR spectra of selected specimens, including the spectrum of an alginate specimen prepared and foamed in the same manner as the tested formulations. The spectrum of raw sodium alginate powder displayed characteristic asymmetric and symmetric stretching bands of carboxylate groups (COO^−^) at 1593 cm^−1^ and 1406 cm^−1^, respectively, which is consistent with documented values [53]. Upon crosslinking, these bands shifted to the 1628–1604 cm^−1^ region, which can be assigned to, and 1427–1419 cm^−1^ regions, respectively, which is consistent with calcium alginate [54]. All spectra exhibit a broad band around 3350–3300 cm^−1^, attributed to stretching vibrations of OH groups in alginate and other hydroxyl-containing components [55,56,57]. The relatively similar positions of COO^−^ stretching bands in chitosan-free AP12 and chitosan-containing AP9Ch and AP12G10Ch specimens indicate that the calcium-crosslinked alginate network remains dominant.

The FTIR spectrum of raw chitosan powder exhibits the following characteristic features: a band at 1590 cm^−1^, associated with N-H vibrations of amino groups, a weak shoulder at 1645 assigned to amide I (C=O stretching), and a band at 1025 cm^−1^ corresponding to the saccharide structure of chitosan [58,59]. In chitosan-containing specimens, a weak shoulder is observed at 1561–1560 cm^−1^. This feature can be associated with N-H bending vibrations, suggesting changes in the local environment of amino groups. The small peak at 2947 cm^−1^, observed in the spectrum of AP12G10Ch, could be assigned to CH stretching vibrations of glycerol [60]. The wide bands in the 1026–1009 cm^−1^ region in the spectra of specimens can be attributed to overlapping contributions from Si-O- stretching vibrations of expanded perlite [61], C-O-C/C-O stretching vibrations of alginate [62], with additional contributions from C-O stretching of chitosan and glycerol in chitosan- and glycerol-containing specimens, respectively [63,64].

### 2.4. Morphological Characterization

**Figure 4:**
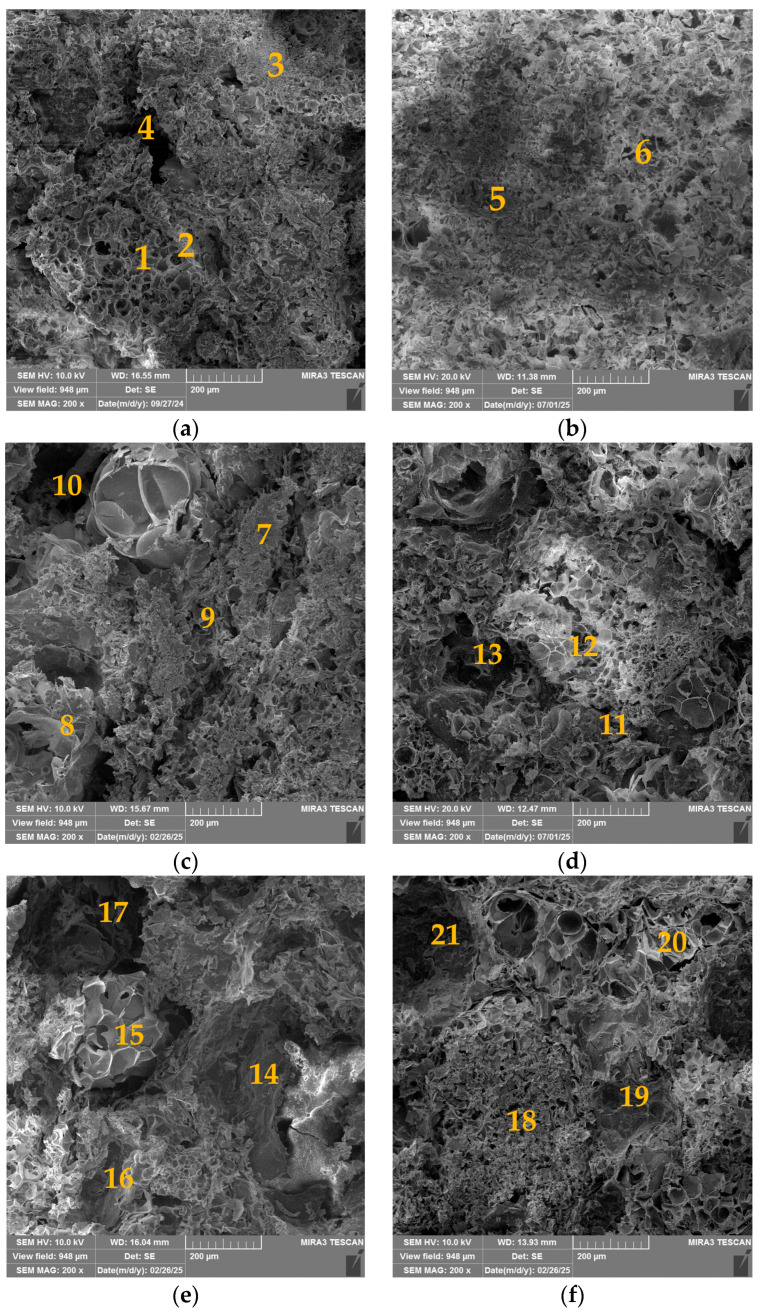
SEM micrographs of cross-section of the specimens, magnification of 200×: (**a**) AP9, (**b**) AP12, (**c**) AP9Ch, (**d**) AP12Ch, (**e**) AP12G5, and (**f**) AP12G10Ch.

SEM micrographs of selected specimens are shown in Figure 4. AP9 (Figure 4a) exhibits an open, permeable structure (1) with expanded perlite particles partially coated by strip-like formations of alginate (2), locally thickened polymer regions (3), and larger interparticle voids (4), resulting in limited polymer bridging and a pronounced open-cell morphology. In AP12 (Figure 4b), particle packing is denser, while the polymer matrix remains thin and discontinuous (5), with exposed particle apices (6).

In AP9Ch (Figure 4c), expanded perlite particles are enveloped by a thicker alginate-chitosan layer with nodular morphology (7), although particle apices remain partially uncovered (8) and exposed particle shells can be observed between adjacent polymer envelopes (9), together with interparticle voids (10). AP12Ch (Figure 4d) shows denser particle packing, with thinner and more uniform polymer coverage (11), partially uncovered apices (12), and smaller interparticle voids conforming to particle geometry (13).

AP12G5 (Figure 4e) is characterized by a relatively continuous and smooth alginate-glycerol film spanning between particles (14), with partially exposed apices (15), locally conformal coatings (16), and interparticle voids containing uncoated particles (17). In contrast, AP12G510Ch (Figure 4f) exhibits a heterogeneous morphology, with irregular, less compact (18) and smoother polymer-covered particle regions (19), exposed perlite shells (20), and cavities (21).

Overall, increasing perlite content shifts the structure from open to more densely packed particle-supported networks, while chitosan improves polymer continuity. Such morphology, characterized by more continuous matrix regions, is expected to influence the mechanical performance of the foams, as discussed in Section 2.6.

### 2.5. Thermal Conductivity and Volumetric Heat Capacity

The thermal conductivity of porous materials comprises four contributions arising from conduction through the solid phase, conduction through the gas phase, convection within the cells, and radiation throughout the cellular structure. Due to the small cell dimensions typically present in cellular materials, natural convection within the cells is generally suppressed, and heat transfer is therefore dominated by conductive and radiative mechanisms [65]. The assessment of radiative heat transfer in porous foam-like materials should involve the consideration of spectral properties of absorption and scattering of infrared radiation within the system [66,67]. In the present study, thermal conductivity was measured by the transient method using an ISOMET 2114 thermal properties analyzer, which analyzes temperature response to heat flow impulses [68]. Detailed spectral characterization of infrared absorption and scattering was beyond the scope of the present work.

**Figure 5:**
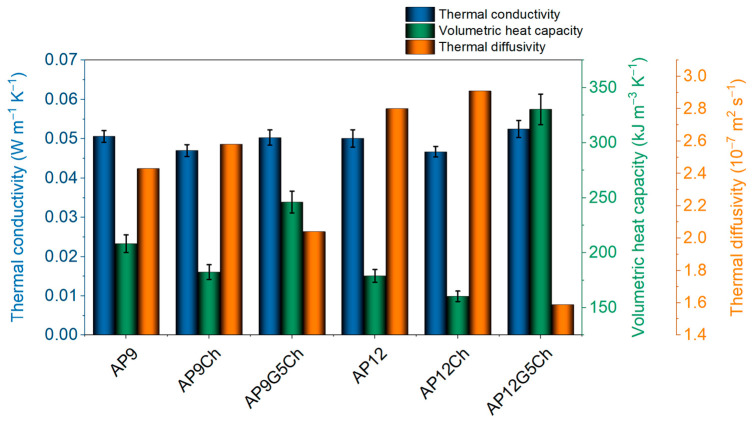
Thermal conductivity, volumetric heat capacity, and thermal diffusivity values of selected tested specimens.

The values of thermal conductivity and volumetric heat capacity, along with the calculated heat diffusivity, are presented in Figure 5. Thermal conductivity values ranged from 0.0467 W m^−1^ K^−1^ (AP9Ch) to 0.0525 W m^−1^ K^−1^ (AP12G5Ch), with the lowest values consistently observed in chitosan-containing glycerol-free formulations. This reduction is attributed to enhanced structural integrity of the pore walls, which suppresses pore coalescence and promotes a more uniform cellular structure. In contrast, volumetric heat capacity values lie within a broader variation (160–330 kJ m^−3^ K^−1^), with the highest values observed in formulations containing glycerol.

Calculated thermal diffusivities clarify the distinct thermal responses of the investigated foams. Although chitosan-containing specimens (AP9Ch and AP12Ch) exhibit the lowest thermal conductivity, their relatively low volumetric heat capacity results in the highest thermal diffusivity, enabling a faster thermal response. In contrast, formulations containing glycerol show the lowest thermal diffusivity values. Despite having slightly higher thermal conductivity, their higher volumetric heat capacity enhances their ability to buffer temperature fluctuations. Consequently, the addition of chitosan primarily improves resistance to heat flow, whereas glycerol contributes to thermal inertia, effectively slowing the propagation of temperature disturbances through the material.

Overall, the measured values of thermal conductivity are within the range of expanded perlite (0.040–0.052 W m^−1^ K^−1^) [69], and are comparable to the values reported for expanded polystyrene (0.031–0.049 W m^−1^ K^−1^) [69,70]. Similar results have been documented for alginate-corn pith composites (0.042 and 0.048 W m^−1^ K^−1^) [71]. While these values are slightly higher than those reported for freeze-dried calcium alginate aerogels (0.030–0.044 W m^−1^ K^−1^) [15,16,19], the investigated foams remain within a comparable range, utilizing a significantly simpler processing route-conventional foaming and drying instead of energy-intensive freeze drying. Furthermore, the volumetric heat capacity of specimens is comparable to conventional polyurethane insulation (~50–250 kJ m^−3^ K^−1^) and rock wool (~50–200 kJ m^−3^ K^−1^), as well as natural fibrous insulators like wood fibers (~50–600 kJ m^−3^ K^−1^) and kenaf (up to ~350 kJ m^−3^ K^−1^) [72]. The synergy of low thermal conductivity and moderate volumetric capacity ensures both effective resistance to heat transfer and the ability to dampen temperature fluctuations, fulfilling essential requirements for building insulation applications.

### 2.6. Mechanical Properties

**Figure 6:**
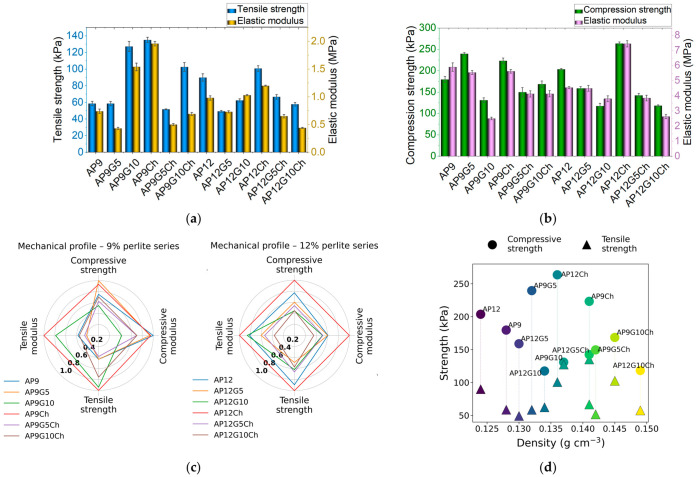
Mechanical properties of alginate-expanded perlite foams: (**a**) tensile strength and elastic modulus, (**b**) compression strength and elastic modulus, (**c**) normalized mechanical profile of the 9 wt.% and the 12 wt.% perlite series (mechanical parameters in radar plots were normalized to the maximum value within each series), and (**d**) comparative overview of mechanical properties.

As shown in Figure 6a, tensile strength spans 49 kPa (AP12G5) to 135 kPa (AP9Ch), while the elastic modulus ranges from 0.44 MPa (AP12G10Ch) to 1.96 MPa (AP9Ch). The superior performance of AP9Ch is consistent with previous results [35], and can be attributed to its microstructure (Figure 4c), which shows more coherent polymer coverage and improved bridging between expanded perlite particles, enabling more efficient stress transfer across the matrix. AP9G10 has the second-highest tensile strength and modulus (127.29 kPa, 1.54 MPa), indicating that, in the 9 wt.% perlite series, increased glycerol content is compatible with good tensile performance. In contrast, the addition of chitosan to high glycerol-containing formulations generally reduces tensile performance, suggesting that the reinforcing role of chitosan is reduced in more strongly plasticized systems. The obtained values fall within the range of conventional insulation materials, with the highest strengths comparable to expanded polystyrene (≥110.3 kPa) [73] and the overall range overlaps with the lower range of rigid polyurethane foams (≥40 kPa) [74] and sodium silicate-bonded expanded perlite systems (~5–150 kPa) [75].

The compressive strength and modulus (Figure 6b) reach maximum values for AP12Ch (263.3 kPa, 7.5 MPa), reflecting a dense perlite framework interconnected by the polymer matrix, which provides effective load-bearing capacity. This response likely arises from the combined contribution of the rigid perlite skeleton and structural reinforcement associated with the presence of chitosan. In contrast, the lowest compressive strength is found for AP12G10 (117.45 kPa) and the lowest modulus (AP9G10, 2.50 MPa), indicating reduced resistance to deformation in formulations with higher glycerol content. A general decrease in compressive strength and modulus with increasing glycerol content can be observed across most formulations. The achieved compressive strengths (~120–260 kPa) are comparable to expanded perlite-sodium silicate composites (~200–300 kPa) [30]. They also overlap with expanded polystyrene insulation materials, corresponding to classes from Cs(10)120 to CS(10)250 that are defined in the EN 13163 standard [21] for expanded polystyrene products used in building insulation applications, also to classes from CS(10/Y)100 to CS(10/Y)250, in the corresponding EN 13164 [22] for extruded polystyrene products. Comparable values are also reported for commercial EPS insulation boards [76].

The radar plots (Figure 6c) highlight that chitosan-only containing formulations exhibit the most balanced mechanical response, combining high tensile and compression strength and modulus. In contrast, glycerol-rich compositions show a less balanced profile, reflecting matrix softening due to plasticization. The paired comparison of 9 wt.% and 12 wt.% perlite shows that perlite content does not have a uniform effect on mechanical performance. For example, the chitosan-only formulation with 9 wt.% perlite gives the highest tensile strength and modulus, while the one with 12 wt.% perlite gives the highest compressive strength and modulus. Thus, the increase in perlite content does not simply improve mechanical properties, but changes which mechanical response is favored. The effect of chitosan also depends on the presence of glycerol. Chitosan is effective in chitosan-only formulations, but it is less effective in glycerol-containing formulations, indicating that its reinforcing role is reduced in plasticized matrices. Increasing glycerol content affects tensile and compression properties differently: generally, tensile properties improve while compressive properties decrease. Therefore, the mechanical behavior reflects the combined effect of perlite, chitosan, and glycerol in the investigated formulations.

Factorial ANOVA supported this interpretation. For tensile strength, the main effects of perlite level, glycerol content, and chitosan addition, as well as their interaction, were significant (*p* < 0.05). For tensile modulus, the main effects of perlite level and glycerol content were significant, while the addition of chitosan was not; however, its interactions with perlite and glycerol were significant. This indicates that chitosan affects modulus mainly through its combination with perlite and glycerol. For compressive strength and modulus, all main effects and interactions were significant. These findings show that the influence of perlite, glycerol, and chitosan depends both on their individual effects and their interactions within the composite formulations. All reported effects were significant at *p* < 0.05.

As shown in Figure 6d, the mechanical performance does not scale directly with density, and given the narrow porosity range (23.9–29.0%), these differences are not governed by porosity alone. Instead, the mechanical response is governed by matrix continuity and the quality of the matrix-particle interface.

**Figure 7:**
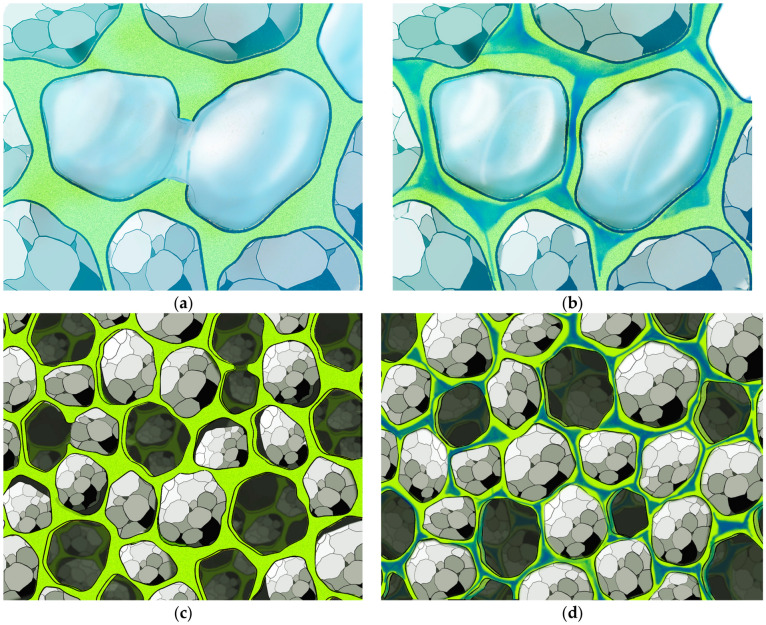
Schematic illustration of the dual role of chitosan in alginate-expanded perlite composites: (**a**) chitosan-free system, bubble coalescence, (**b**) chitosan-containing system, suppressed coalescence, (**c**) chitosan-free system with matrix discontinuities, (**d**) chitosan-containing system with enhanced matrix continuity. Green regions represent alginate matrix, while blue shaded regions schematically indicate chitosan-associated reinforcement zones. The illustration is intended only to visualize the proposed reinforcing effect of chitosan.

Overall, chitosan appears to have a dual purpose in the composites investigated. First, during foaming, chitosan particles act as physical barriers that prevent bubbles from coalescing (Figure 7a,b). This “pore-refining” effect probably contributes to the stabilization of the wet foam and the formation of a more uniform cellular structure. Second, after cross-linking and drying, chitosan could contribute to the reinforcement of alginate struts between expanded perlite particles, and to improved matrix continuity (Figure 7c,d). This structural contribution is consistent with more effective stress transfer across the composite. Taken together, the results suggest that chitosan and glycerol influence different aspects of foam structure and performance, with chitosan mainly associated with pore refinement and structural connectivity, and glycerol related mainly to flexibility and thermal inertia.

### 2.7. Water Absorption

**Figure 8:**
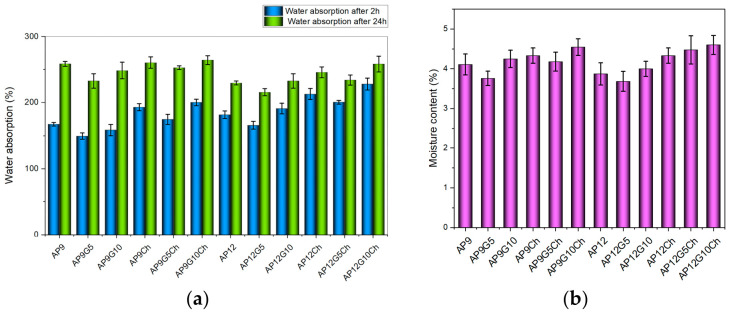
(**a**) Water absorption of specimens after immersion in water for 2 h and 24 h, (**b**) moisture content of specimens.

Figure 8a presents the water absorption of the specimens after 2 and 24 h of immersion. As expected, uptake increased over time, though formulations with 5 wt.% glycerol consistently exhibited the lowest values. Minimum absorption was observed for AP9G5 (159.8% at 2 h and 227.8% at 24 h) and AP12G5 (165.8% at 2 h and 220.3% at 24 h). SEM micrograph of AP12G5 (Figure 4e) reveals a relatively smooth and compact polymer layer with fewer openings, suggesting that moderate glycerol content may limit water ingress.

In contrast, the highest water absorption values are observed for specimens containing both chitosan and glycerol. The AP9G10Ch specimen showed the maximum uptake (264.0% at 2 h and 269.9 wt.% at 24 h), followed by AP9Ch (260.3% at 24 h). SEM observations of AP9Ch (Figure 4c) show continuous gaps and exposed perlite shells, providing direct channels for water penetration. These results indicate that chitosan incorporation increases water uptake, particularly when combined with glycerol. Absorption is promoted due to the hydrophilic nature of both additives. Additionally, specimens with 12 wt.% perlite generally exhibited lower uptake than those with 9 wt.%, as the higher filler fraction reduces the proportion of the hydrophilic matrix. Despite the higher water uptake, all specimens maintained structural integrity with dimensional changes not exceeding 1.4%.

While these water absorption values significantly exceed those of commercial polymeric insulation foams [77,78,79], water absorption is not a critical drawback for the intended applications. These materials are designed for use within the building envelopes (wall or roof cavities), shielded from direct atmospheric moisture. To further enhance water resistance in future iterations, surface modifications of expanded perlite, such as silane-based or fatty acid hydrophobic coatings, could be implemented [80,81].

### 2.8. Moisture Content

The results of the moisture content measurement are given in Figure 8b. The values ranged from 3.68 to 4.98% across the investigated formulations. Within both perlite-content groups, the highest moisture content is observed for the formulations with 10 wt.% glycerol. Regarding the triplet groups with formulations containing different glycerol levels, formulations with 5 wt.% glycerol generally had the lowest values.

### 2.9. Flammability Test Results

**Figure 9:**
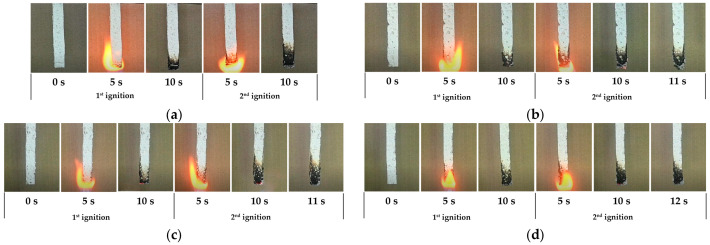
Behavior of exemplary specimens during the vertical flammability test: (**a**) AP9Ch specimen with no afterglow observable, and specimens with afterglow observable after both ignitions: (**b**) AP12, (**c**) AP12G10, and (**d**) AP12G5Ch.

Digital photographs from the vertical flammability test (Figure 9) demonstrate that all investigated composites exhibit self-extinguishing behavior. During flame exposure, the materials did not develop a propagating burning front, and no flames remained upon burner removal. Residual afterglow was minimal, lasting 0–2 s depending on the formulation, with chitosan-only specimens showing no afterglow after both ignition cycles. Furthermore, no burning droplets were formed, ensuring the integrity of the cotton indicator (see Supplementary Information, Figure S2).

Based on these results, all specimens are classified under the V-0 rating. This performance significantly distinguishes the developed foams from unmodified expanded polystyrene and rigid polyurethane, which typically do not show comparable UL-94 performance, being reported as V-2 or not classified under the test conditions in the literature [5,6].

The flame-retardant performance is attributed to the synergistic effect of the primary constituents. Calcium alginate promotes a condensed-phase mechanism through char formation [38], while expanded perlite acts as a thermally stable mineral barrier that limits heat transfer and contributes to the physical shielding of the underlying material [82,83,84]. In addition, chitosan may contribute to stabilization of the protective residue, as indicated by the absence of afterglow in chitosan-containing specimens. Together, these components contribute to the formation of a mineral-char protective layer that hinders further combustion.

## 3. Conclusions

The present study demonstrates that lightweight alginate-expanded perlite foams can be fabricated via in situ CO_2_ foaming and Ca^2+^ crosslinking, followed by conventional mild temperature drying. This simple, water-based processing route enables the preparation of alginate-based xerogel foam materials without the need for freeze-drying. The resulting materials exhibited low densities (0.124 to 0.149 g cm^−3^), surface porosity in the range of 23.9–29.0%, thermal conductivities in the range of 0.0467–0.0525 W m^−1^ K^−1^, and UL-94 V-0 self-extinguishing behavior. Mechanical performance is strongly governed by composition. The incorporation of 1 wt.% chitosan improves both tensile (135 kPa) and compressive strength (263 kPa). Introducing chitosan in dispersed form, rather than by acidic solution, promotes controlled foaming and homogeneous distribution of expanded perlite particles, consistent with improved matrix continuity and particle coverage.

Overall, these results demonstrate that alginate-expanded perlite composites possess mechanical performance suitable for self-supporting insulation applications. The low thermal conductivity, self-extinguishing behavior, use of abundant and benign constituents, and scalability support the potential of alginate-expanded perlite foams as a practical alternative to fossil-derived insulation materials. Future work should focus on tailoring pore architecture and reducing water uptake to further optimize the balance between mechanical, thermal, and moisture-related properties.

## 4. Materials and Methods

### 4.1. Materials

Sodium alginate (food grade, E401) was purchased from Würzteufel GmbH (Empfingen, Germany). Chitosan (food grade) was sourced from Mystic Moments (Fordingbridge, UK). Expanded perlite, type P2, was sourced from Termika d.o.o., (Zrenjanin, Serbia) [27]. General properties of the expanded perlite and the particle size distribution of the specific batch used in this study are provided in Supplementary Information (Tables S1 and S2, respectively). Sodium bicarbonate was obtained from Zorka Pharma-Hemija d.o.o. (Šabac, Serbia). Glycerol, acetic acid, and calcium chloride were purchased from BetaHem d.o.o. (Belgrade, Serbia).

### 4.2. Preparation of Specimens

**Figure 10:**
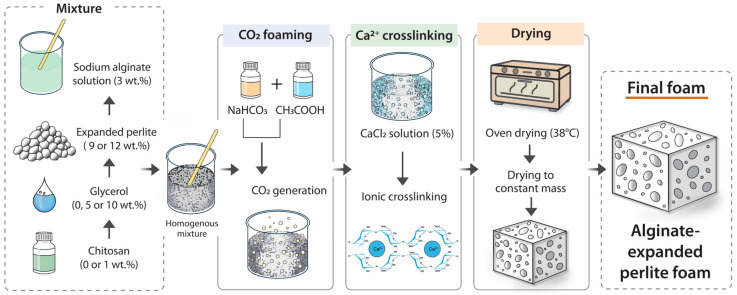
Schematic representation of the processing route for alginate-expanded perlite foams.

A preliminary foaming screen (5–15 wt.% expanded perlite) showed that mixtures below 8 wt.% perlites were too fluid and spread even without foaming agents, whereas those above 14 wt.% were too viscous, leading to particle segregation. Based on those observations, 12 formulations were selected and prepared following a modified procedure from a previous study [35] (Figure 10). Components were sequentially added to the sodium alginate solution in distilled water (Table 1). Foaming was achieved via in situ CO_2_ generation using sodium bicarbonate and pouring the mixture over a 20 vol.% acetic acid solution, according to:

CH_3_COOH + NaHCO_3_ = CH_3_COONa + H_2_O + CO_2_ (1)

The mixture was stirred (90 rpm), followed by a 75 s resting step to allow an undisturbed gas-forming reaction and a more uniform pore structure. After the mixture was transferred to molds, a 5 wt.% CaCl_2_ solution in distilled water was gradually added to induce ionic crosslinking. In chitosan-free formulations, bubble coalescence and upward migration were observed; this was mitigated by manual mold rotation for 2 min after the CaCl_2_ addition. Specimens were left undisturbed for 24 h, air-dried for 24 h, rinsed with distilled water, and oven-dried at 38 °C to constant mass. The temperature was chosen to promote the removal of water while maintaining the structural integrity of the matrix and preventing pore distortion and collapse. Calculated dry-state compositions after crosslinking and drying are given in Table 2, while formulation labels in the text refer to initial wet-state compositions.

Chitosan is typically dissolved in diluted acids [85,86]; however, this approach was not used here, as mixing dissolved chitosan with alginate solution resulted in immediate gelation, consistent with the formation of a polyelectrolyte complex driven by ionic interactions between COO^−^ groups of alginate and protonated -NH3^+^ groups of chitosan [87]. Such behavior hindered the uniform dispersion of expanded perlite particles and disrupted the foaming process. Preliminary tests confirmed that mixing of dissolved chitosan and alginate caused partial gelation and produced structurally unstable or delaminated specimens after crosslinking and drying. Although the interaction strength and formation of alginate-chitosan polyelectrolyte complexes depend on conditions such as pH [59], such modifications would introduce additional process sensitivity and complicate the simultaneous control of dispersion and foaming.

**Figure 11:**
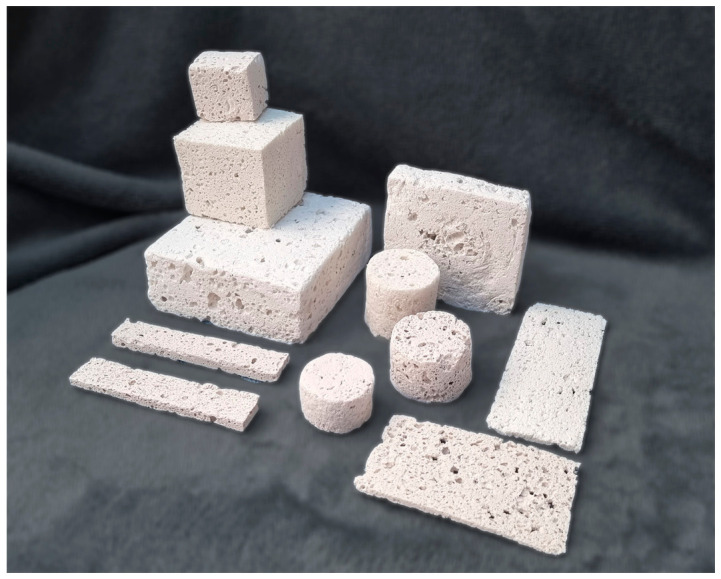
Specimen forms prepared for characterization procedures and evaluation of potential product applications.

In the absence of chitosan, repeated inversion of molds after CaCl_2_ addition was required to suppress bubble growth and improve pore uniformity. Representative specimen shapes obtained during optimization of compositions and processing conditions are shown in Figure 11.

### 4.3. Characterization

Scanning electron microscopy (SEM), Fourier transform infrared spectroscopy (FTIR), image analysis, diameter shrinkage, thermal conductivity and heat capacity measurements, mechanical testing (compression and Brazilian test), water absorption, moisture content, and flammability testing were used to characterize the specimens. For diameter shrinkage measurements, water absorption test, moisture content measurement, thermal properties, and mechanical properties measurements, three specimens were prepared for each formulation. Statistical evaluation of pore and size data was performed using factorial analysis of variance (ANOVA). Detailed experimental procedures are provided in Supplementary Information S4.3 [27,69,72,88,89,90,91,92,93,94,95,96].

Generative AI use. ChatGPT o3 (OpenAI) was used for code generation in Python 3.8.17 during the development of this work. Specifically, AI-assisted programming support was employed to streamline routine coding tasks and improve efficiency. All generated code was subsequently reviewed, validated, and tested by the authors to ensure accuracy, reproducibility, and compliance with the scientific and ethical standards of the journal.

## Figures and Tables

**Table 1:** Initial compositions of specimens prior to foaming.

Specimen	Component Weight Ratio, wt.%				
	Sodium Alginate	Perlite	Chitosan	Glycerol	Water
AP9	3	9	/	/	88
AP9G5	3	9	/	5	83
AP9G10	3	9	/	10	78
AP9Ch	3	9	1	/	87
AP9G5Ch	3	9	1	5	82
AP9G10Ch	3	9	1	10	77
AP12	3	12	/	/	85
AP12G5	3	12	/	5	80
AP12G10	3	12	/	10	75
AP12Ch	3	12	1	/	84
AP12G5Ch	3	12	1	5	79
AP12G10Ch	3	12	1	10	74

**Table 2:** Calculated compositions of specimens after foaming, crosslinking, and drying.

Specimen	Component Weight Ratio, wt.%			
	Sodium Alginate	Perlite	Chitosan	Glycerol
AP9	24.7	75.3	0.0	0.0
AP9G5	17.4	53.1	0.0	29.5
AP9G10	13.5	41.0	0.0	45.5
AP9Ch	22.8	69.5	7.7	0.0
AP9G5Ch	16.5	50.1	5.6	27.8
AP9G10Ch	12.9	39.2	4.4	43.5
AP12	19.8	80.2	0.0	0.0
AP12G5	14.8	59.9	0.0	25.3
AP12G10	11.9	48.1	0.0	40.0
AP12Ch	18.5	75.1	6.4	0.0
AP12G5Ch	14.1	57.2	4.9	23.8
AP12G10Ch	11.4	46.2	4.0	38.4

## Data Availability

The data presented in this study are available on request from the corresponding authors. The data are not publicly available.

## Supplementary Materials

The following supporting information can be downloaded at https://www.mdpi.com/article/10.3390/gels12070625/s1, Figure S1: Schematic representation of stress zones during the Brazilian split test: (1) zone of triaxial stress, (2) zone of uniaxial stress, (3) neutral zone, and (4) tensile stress zone; Figure S2: Setup illustration for vertical UL-94 tests; Table S1: Expanded perlite properties; Table S2: Granulometric distribution of expanded perlite type P2; Table S3: Classification of materials according to the UL-94 testing results. The references cited in the Supplementary Materials correspond to the references [27,69,72,88,89,90,91,92,93,94,95,96] in the main manuscript.
