# Supplementary material for: Self-Extinguishing Alginate-Based Xerogel Foams for Thermal Insulation
Source: Gels. 2026 Jul 11;12(7):625. doi: 10.3390/gels12070625 (PMC13409490; doi:10.3390/gels12070625)
Supplement: Supplementary file 1 [file gels-12-00625-s001.zip › gels-4408987-supplementary.pdf]

Supplementary data

S4.1. Materials

Table S1. Expanded perlite properties [1–3]

Bulk density, kg m^{-3}	Thermal conductivity, W m^{-1} K^{-1}	Specific heat capacity, J kg^{-1} K^{-1}	Water absorption, %
31–176	0.040–0.052	0.9–1.1	200–800

Table S2. Granulometric distribution of expanded perlite type P2 [4]

Parameters	Sieves diameter (mm)	Average value
Granulometric content	+ 1.000	11.4
	-1.000 + 0.315	52.8
	-0.315 + 0.200	12.7
	-0.200	23.1

4.3. Characterization

Diameter shrinkage values were calculated as diameter after drying per diameter of wet soaked off specimen after removal from the $CaCl_2$ bath. The apparent density values of specimens were calculated as mass per unit volume.

Image analysis was performed in Python 3.8.17 programming language and NumPy 1.21.2, Matplotlib 3.7.1 and OpenCV 4.6.0 image processing libraries. For each specimen, images acquired under opposite lighting were centrally cropped to an identical region of interest (ROI) and fused by pixel-wise averaging to reduce lighting-induced artifacts. The fused images were converted to grayscale, processed using contrast-limited adaptive histogram (CLAHE), and smoothed by Gaussian filtering. Segmentation was performed using global Otsu thresholding, resulting in binary classification of pore (black) and solid matrix (colored) pixels. Surface porosity was calculated as the ratio of pore pixels to total pixels within ROI. Individual pores were identified as connected components in the binary images, and their size was expressed as equivalent circular diameter, calculated from the measured pore area after pixel-to-mm calibration. The obtained pore diameter datasets were used to construct pore size distributions and to calculate percentile descriptors D10, D50, and D90, corresponding to the 10%, 50% and 90% percentiles, respectively. Percentile values were calculated using NumPy 1.21.2, tabulated and summarized using Pandas 1.5.3, while pore size distribution curves were generated using Gaussian kernel density estimation in SciPy 1.10.1 and plotted using Matplotlib 3.7.1.

Fourier transform infrared (FTIR) analysis was performed using NicoletTM iSTM 10 FT-IR spectrometer (Thermo Fisher SCIENTIFIC, Waltham, MA, USA) with Smart iTRTM Attenuated Total Reflectance (ATR) sampling accessories. FTIR spectra was measured in 20 scan modes in the range of 4000–500 cm^{-1} with a 4 cm^{-1} resolution. Microstructures of samples were examined using a 20kV emission scanning electron microscope (FE-SEM Mira3 Tescan, Oxford, UK). A small layer of Au was sputtered on the sample prior to imaging.

Measurement of thermal conductivity and volumetric heat capacity was performed on ISOMET 2114 on specimens with size 10×10×5 cm. For each composition three specimens were tested, and average thermal conductivity and volumetric heat capacity presented. In addition, thermal diffusivity was calculated using the following equation [5]:

$$\alpha = \lambda / C_v \tag{S1}$$

where α is thermal diffusivity ($m^2\ s^{-1}$), λ thermal conductivity ($W\ m^{-1}\ K^{-1}$) and C_v volumetric heat capacity ($J\ m^{-3}\ K^{-1}$).

Compression and Brazilian tests were conducted using Instron dynamometer (model 1185) testing machine, equipped with a 10 kN load cell with crosshead speed of 5 mm/min at room temperature (23±1 °C) and 51±3 % relative humidity. Regarding the tensile testing, to minimize potential gripping and standardized specimen geometry challenges for brittle materials, indirect tensile strength was determined using the Brazilian test, according to the standard SRPS B.B7.127:2020 [6], using specimens with diameter-to-thickness ratio 2:1. For compression testing general principles of EN 826 [7] and ISO 844 [8] were employed as a guideline; to ensure consistency with the dimensional requirements of the standards, specimens were prepared with height-to-width ratio 1:1. For both mechanical tests three specimens of each composition were tested and calculated mean values were presented. The stress distribution zones during the Brazilian test are illustrated in Figure S1.

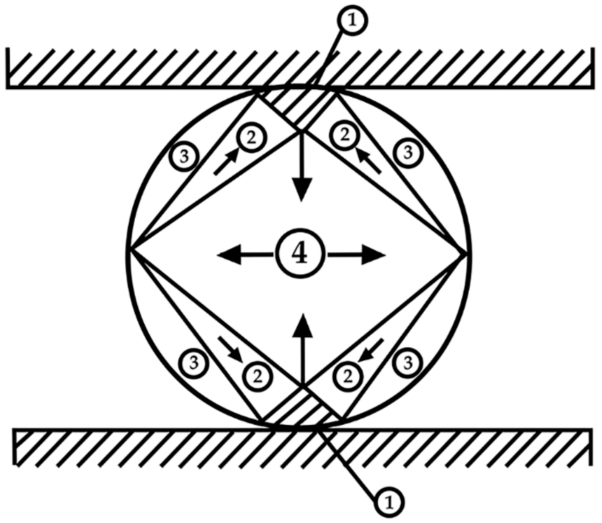

Figure S1. Schematic representation of stress zones during the Brazilian split test: 1) zone of triaxial stress, 2) zone of uniaxial stress, 3) neutral zone, and 4) tensile stress zone

For the water absorption test, three disk-shaped specimens (of the same sizes used for mechanical testing) of each of the twelve compositions were dried to constant mass. One set was immersed in water for 2 hours and the other one for 24 hours. In both cases, after the removal from water, the specimens were carefully blotted to remove surface water. Water uptake was calculated according to the formula:

$$WU\ (100\%) = 100 * (M_1 - M_0)/M_0 \tag{S2}$$

where WU is water uptake after 2 h (or 24 h) immersion, M_0 mass of initial dry specimen, and M_1 is mass of specimen after 2 h (or 24 h).

The moisture content was determined using a procedure based on the principles of ISO 12571 [9] and ASTM D5229 [10]. For each formulation three disk-shaped specimens (of the same sizes used for mechanical testing) were dried to constant mass at 40°C; higher drying temperatures could cause loss of glycerol in specimens where it was contained, as reported in literature [11]. After drying, specimens were exposed to relative humidity of 50% at room temperature at 25°C for 24 h until equilibrium moisture content (EMC) was reached. The equilibrium moisture content was calculated according to the following equation:

$$EMC\ (\%) = 100 * (M_{eq} - M_0)/M_0 \quad (S3)$$

where M_0 is mass of dried specimen, and M_{eq} mass of specimen after conditioning at 25°C and 50% RH until equilibrium was reached.

A standardized flame test, based on the UL-94V procedure [12], was conducted to assess the flammability of the composite materials investigated. Details of the test criteria are provided in Table S3. The specimens were rectangular, with dimensions of 10 mm in length, 10 mm in width, and 5 mm in thickness. They were mounted vertically, and a 20 mm high blue flame (Messer, Serbia) was applied to the lower end of each specimen for 10 s. Upon flame removal, the time required for the specimen to self-extinguish was recorded. The flame was then reapplied for another 10 s, and the self-extinguishing time was recorded again. A cotton pad was placed beneath the fixture to observe any potential ignition by burning or molten droplets, in accordance with UL-94V classification criteria. A schematic of the experimental setup is provided in Figure S2.

Table S3. Classification of materials according to the UL-94 testing results [12]

**Criteria conditions**	**V-0**	**V-1**	**V-2**
After flame time for each individual flaming	≤10s	≤30s	≤30s
After flame and glow time for each individual specimen, after the second flaming	≤30s	≤60s	≤60s
Total after flame time for any condition set	≤50s	≤250s	≤250s
Cotton indicator ignited by flaming drops	No	No	Yes
After flame and afterglow time of any specimen up to the holding clamp	No	No	No

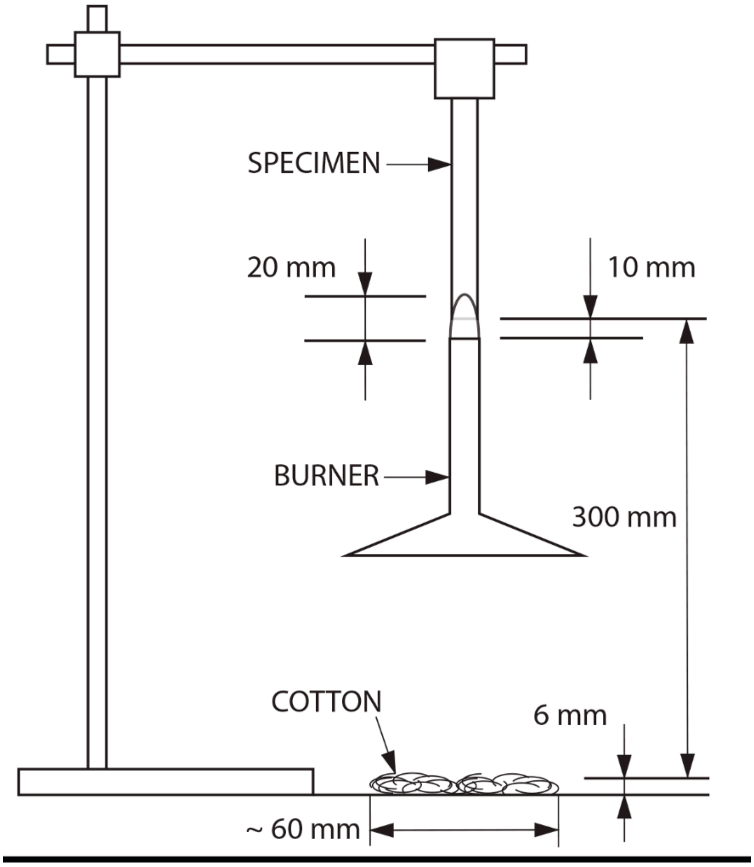

Figure S2. Setup illustration for vertical UL-94 tests

Statistical analysis was performed in Python 3.8.17 programming language using NumPy 1.21.2, Pandas 1.5.3, SciPy 1.10.1 and statsmodel 0.13.5 libraries. Factorial analysis of variance (ANOVA) was used to evaluate the effects of perlite level, glycerol content and chitosan addition, and their interactions on each measured mechanical property. For pore size descriptors, factorial ANOVA was applied to both original and log-transformed D10, D50 and D90 values. Log-transformed pore size data were additionally considered because of the right-skewed nature of pore size distribution. Effects were considered statistically significant at $p < 0.05$.

References

1. Schiavoni, S.; D'Alessandro, F.; Bianchi, F.; Asdrubali, F. Insulation Materials for the Building Sector: A Review and Comparative Analysis. *Renew. Sustain. Energy Rev.* **2016**, *62*, 988–1011, doi:10.1016/j.rser.2016.05.045.

2. Kumar, D.; Alam, M.; Zou, P.X.W.; Sanjayan, J.G.; Memon, R.A. Comparative Analysis of Building Insulation Material Properties and Performance. *Renew. Sustain. Energy Rev.* **2020**, *131*, 110038, doi:10.1016/j.rser.2020.110038.

3. Davraz, M.; Koru, M.; Isildar, N. Design of Perlite Based Thermal Insulation Plate and

Determination of Its Physical, Mechanical and Thermal Properties. *Int. J. Thermophys.* **2025**, *46*, 31, doi:10.1007/s10765-025-03503-x.

4. Termika d.o.o. *PERLIT P-2 Product Declaration*; 2024; Available online: https://www.termika.rs/perlit/specifikacije/perlit_p2.pdf (accessed on 26 April 2025)

5. Bergman, T.L..; Levine, A.S.. *Fundamentals of Heat and Mass Transfer*; John Wiley & Sons, Inc., 2019; ISBN 1119320429.

6. *SRPS B.B7.127:2020 Rock Mechanics—Testing of Physical and Mechanical Properties—Method for the Determination of Tensile Strength Limit—Indirect Method*; Institute for Standardization of Serbia: Belgrade, 2020.

7. *EN 826: Thermal Insulating Products for Building Applications — Determination of Compression Behaviour*; 2013.

8. *ISO 844:2007 Rigid Cellular Plastics — Determination of Compression Properties*; International Organization for Standardization (ISO), 2007.

9. *ISO 12571 Hygrothermal Performance of Building Materials and Products – Determination of Hygroscopic Sorption Properties*; 2021; Vol. 2021.

10. *ASTM D5229/D5229M-20 Standard Test Method for Moisture Absorption Properties and Equilibrium Conditioning of Polymer Matrix Composite Materials*; 2012.

11. Silva, M.A. da; Bierhalz, A.C.K.; Kieckbusch, T.G. Influence of Drying Conditions on Physical Properties of Alginate Films. *Dry. Technol.* **2012**, *30*, 72–79, doi:10.1080/07373937.2011.620727.

12. American National Standards Institute.; Underwriters' Laboratories. *UL 94: Standard for Tests for Flammability of Plastic Materials for Parts in Devices and Appliances.*; 2001; ISBN 0762900822.
